# Long‐term survivors with desmoplastic small round cell tumor (DSRCT): Results from a retrospective single‐institution case series analysis

**DOI:** 10.1002/cam4.5829

**Published:** 2023-03-23

**Authors:** Claudia Giani, Stefano Radaelli, Rosalba Miceli, Lorenza Gandola, Claudia Sangalli, Anna Maria Frezza, Salvatore Provenzano, Sandro Pasquali, Rossella Bertulli, Marco Fiore, Dario Callegaro, Michela Casanova, Stefano Chiaravalli, Paola Collini, Gian Paolo Dagrada, Carlo Morosi, Nadia Zaffaroni, Paolo G. Casali, Andrea Ferrari, Alessandro Gronchi, Silvia Stacchiotti

**Affiliations:** ^1^ Department of Medical Oncology Fondazione IRCCS Istituto Nazionale dei Tumori Milan Italy; ^2^ Department of Surgery Fondazione IRCCS Istituto Nazionale dei Tumori Milan Italy; ^3^ Department of Clinical Epidemiology and Trial Organisation Fondazione IRCCS Istituto Nazionale dei Tumori Milan Italy; ^4^ Pediatric Radiotherapy Unit Fondazione IRCCS Istituto Nazionale dei Tumori Milan Italy; ^5^ Department of Radiation Therapy Fondazione IRCCS Istituto Nazionale dei Tumori Milan Italy; ^6^ Department of Applied Research and Technological Development Fondazione IRCCS Istituto Nazionale dei Tumori Milan Italy; ^7^ Department of Pediatric Oncology Fondazione IRCCS Istituto Nazionale dei Tumori Milan Italy; ^8^ Department of Diagnostic Pathology and Laboratory Medicine Fondazione IRCCS Istituto Nazionale dei Tumori Milan Italy; ^9^ Department of Radiology Fondazione IRCCS Istituto Nazionale dei Tumori Milan Italy; ^10^ Medical Oncology Università degli Studi Milan Italy

**Keywords:** chemotherapy, prognosis, prognostic factors, radiotherapy, sarcoma, surgery, survival

## Abstract

**Objective:**

To report on a retrospective study of primary DSRCT aiming at characterizing long‐term survivors (LTS).

**Methods:**

All consecutive patients treated at our institution for a primary DSRCT between 2000 and 2021 were retrospectively identified. Patients received multiagent chemotherapy ± surgery ± hyperthermic intraperitoneal chemotherapy (HIPEC) ± whole abdomino‐pelvic radiotherapy (WAP‐RT) ± high‐dose chemotherapy ± maintenance chemotherapy (MC). Event‐free survival (EFS) and overall survival (OS) were estimated by Kaplan–Meier method. Patients alive, without evidence of disease at ≥36 months from diagnosis, were defined as LTS.

**Results:**

Thirty‐eight patients were identified. All received multiagent chemotherapy; 27/38 (71%) surgery (7/27 [26%] plus HIPEC), 9/38 (24%) WAP‐RT, 12/38 (32%) MC. At a median‐follow‐up of 37 months (IQR 18–63), overall median‐EFS and median‐OS were 15 and 37 months, respectively. All events occurred within 35 months. In patients who underwent surgery, median‐EFS and median‐OS were 19 and 37 months (23 and 43 months after R0/R1, and 10 and 19 months after R2 resection), respectively. LTS were 5/38 (13%), alive at 37, 39, 53, 64, 209 months. None had liver or extra‐abdominal metastasis at diagnosis, they all received R0/R1 resection, 3/5 had WAP‐RT, 2/5 MC, 1/5 received high‐dose chemotherapy, none HIPEC.

**Conclusions:**

In our series cure was likely achieved in 13% of DSRCT. LTS had no liver/extra‐abdominal disease, were treated with complete surgery, and possibly WAP‐RT/MC.

## Graphical abstract

‐ DSRCT patients showed a poor outcome, with long‐term event‐free survival of 15%‐ However, a few long‐term survivors (LTS) could be identified (5/38, 13%) ‐ All LTS had no extra‐peritoneal disease and were completely resected

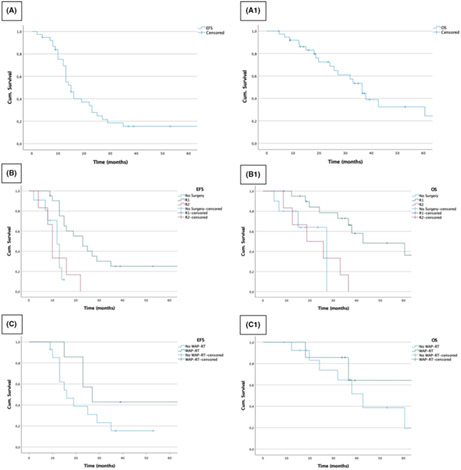

## 1 INTRODUCTION

Desmoplastic small round cell tumor (DSRCT) is a high‐grade ultra‐rare soft tissue sarcoma (incidence 0.2/1,000,000/year),^1^, ^2^ characterized by the presence of the *EWSR1‐WT1* fusion gene.^3^, ^4^, ^5^ DSRCT mostly affects male adolescents and young adults and usually presents with intra‐abdominal multiple nodules originating from the peritoneal surface; it is frequently metastatic at diagnosis and the most common sites of metastasis are lymph nodes (LN), liver, and lungs.^3^, ^4^, ^6^, ^7^

Currently, there is no standard treatment for patient with DSRCT, even if an aggressive multimodality approach is commonly used strategy. This is based on results derived only from retrospective and often conflicting series. Primary tumor treatment includes multiagent chemotherapy, complete cytoreductive surgery and, in some cases, post‐operative whole abdominopelvic radiotherapy (WAP‐RT) and maintenance chemotherapy (MC).^8^, ^9^, ^10^, ^11^, ^12^ Hyperthermic peritoneal perfusion with chemotherapy (HIPEC) can be combined to a macroscopically complete resection.^13^ Other attempted strategies are high‐dose chemotherapy with stem cell rescue.^14^, ^15^ Unfortunately, the vast majority of patients ultimately relapse and die of disease, with a reported median overall survival (OS) in the range of 17–60 months.^3,8–11,16–18^ A minority of patients are long‐term survivors (LTS), although their features have not been characterized yet (Table SS1).

Herein, we report the results of a retrospective analysis of patients affected by primary DSRCT treated at Fondazione IRCCS Istituto Nazionale Tumori (Milan, Italy) (INT), starting from 2000, aiming to define the proportion of LTS, to describe their characteristics and identify predictors of a better outcome.

## 2 METHODS

All consecutive patients of any age, treated at INT between January 2000 and December 2021 for primary DSRCT, were retrospectively identified. Diagnosis was reviewed according to the WHO classification,^5^ and confirmed in all cases by the presence of *EWSR1‐WT1* fusion investigated by molecular and/or immuno‐histochemical assessment.

Data were extracted from a prospectively maintained institutional database and confirmed through a review of patient files. All patients provided a written informed consent to their treatment. Approval by the institutional review board was obtained.

### 2.1 Treatment

All patients were evaluated within the institutional multidisciplinary tumor board. Their treatment consisted of a multimodal approach including multiagent chemotherapy, cytoreductive surgery ± WAP‐RT. Patients were treated according to different multi‐agent chemotherapy regimens, all containing anthracyclines and ifosfamide. Three weeks of treatment was counted as one cycle. Data on MC (Table S2) or high‐dose chemotherapy were also collected. MC was administered on a case‐by‐case basis following the first‐line treatments until progression/unacceptable toxicity for a maximum of 12 months. Response to first‐line chemotherapy was retrospectively assessed by RECIST 1.1.^19^

Cytoreductive surgery was performed when technically feasible and combined with HIPEC using cisplatin upon decision of the multidisciplinary tumor board. HIPEC was performed mostly in case of disseminated peritoneal involvement and surgery was not supposed to be macroscopically complete. Peritoneal cancer index (PCI) was not used as selection criteria for surgery and/or HIPEC in our series. Surgery was classified as macroscopically complete (R0/R1) or incomplete (R2). Postoperative WAP‐RT was mostly prescribed in pediatric patients, when feasible (Table S3). Total radiation dose (range between 15 and 32 Gy, with a median dose of 25.5 Gy) was based on the radiation techniques available during the study period (three‐dimensional conformal vs. volumetric modulated arc therapy‐VMAT) WAP‐RT delivered a high radiation dose to the whole abdomen respecting dose constraints for the kidney and the liver. The kidneys were shielded after 12–15 Gy and the liver after 25–26 Gy. Treatment was delivered with fractions of 1.5 Gy.

### 2.2 Statistical analysis

Patients were stratified based on disease extent at diagnosis: single mass, multiple peritoneal nodules, peritoneal nodule(s) plus LN involvement, liver metastases, supra‐diaphragmatic disease.

Event‐free survival (EFS) was defined as the interval from the date of diagnosis to that of disease relapse, first progression or death, whichever occurred first, or last follow‐up for patients without events. OS time was defined as the interval from the date of diagnosis to that of death or last follow‐up. EFS and OS curves were estimated by the Kaplan–Meier method. The outcome comparison between patients treated versus not treated with WAP‐RT was performed without conducting any statistical testing due to the limited number of the first subgroup. However, we conducted a matched‐paired analysis by pairing each WAP‐RT‐treated patient with untreated patient matched by disease extent at diagnosis and the surgical resection. Patients alive and with no evidence of disease at ≥36 months from diagnosis were defined as LTS. Statistical analyses were performed using IBM SPSS 27.

## 3 RESULTS

Between 2000 and 2021, 38 consecutive patients with primary DSRCT were retrospectively identified. At the time of diagnosis, the median age was 25 years (range 7–64). Table 1 summarizes patient characteristics and treatments. Only 12/38 (32%) patients presented with peritoneal disease alone.

**TABLE 1:** Patient characteristics.

Patient	
*Gender*	
Female	6/38 (16%)
Male	32/38 (84%)
*Median age* (*years*)	25 (range 7–64)
*Disease extent at diagnosis*	
Peritoneal disease only	12/38 (32%)
Single mass	5/12 (42%)
Multiple peritoneal nodules	7/12 (58%)
Peritoneal disease + sub‐diaphragmatic LN	10/38 (26%)
Liver metastases	8/38 (21%)
Supra‐diaphragmatic disease	8/38 (21%)
*Treatment*	
Multiagent CT	38/38 (100%)
Surgery	27/38 (71%)
+ HIPEC	7/27 (26%)
WAP‐RT	9/38 (24%)
High‐dose CT	5/38 (13%)
Maintenance CT	12/38 (32%)
*Response to first‐line CT*	
Not evaluable	8/38 (21%)
Evaluable	30/38 (79%)
Partial response	16/30 (54%)
Stable disease	13/30 (43%)
Progressive disease	1/30 (3%)
*Surgical margins*	
R0/R1 resection	21/27 (78%)
+WAP‐RT	WAP‐ RT in 7/21 (33%)
R2 resection	6/27 (22%)
+WAP‐RT	WAP‐RT in 2/6 (33%)

Abbreviations: CT, chemotherapy; HIPEC, hyperthermic intraperitoneal chemotherapy; LN, lymph nodes; WAP‐RT, whole abdomino‐pelvic radiation therapy.

All 38 patients received multiagent chemotherapy with different regimens (Table S4), for a median of eight cycles (range 2–10). Thirty of 38 patients were evaluable for response (7/38 treated with adjuvant chemotherapy; 1/38 response assessment not available), among which 16 (54%) achieved a partial response (PR), 13 (43%) had stable disease (SD), and one (3%) progressed.

Twenty‐seven of 38 (71%) patients underwent surgery, while 11/38 (29%) had inoperable disease. Among resected patients, 21/27 (78%) had a R0/R1 and 6/27 (22%) a R2 resection. Nine of 27 (33%) patients received WAP‐RT, 7/21 (33%) after a R0/R1 resection while 2/6 (33%) after a R2 resection; 7/27 (26%) patients received HIPEC, combined to R0/R1 resection in 5/7 (71%) cases, R2 in 2/7 (29%). Eight of 27 (30%) patients received MC, 7/8 (88%) after R0/R1 resection (preceded by WAP‐RT in 5/7), 1/8 (12%) after R2 resection and WAP‐RT. High‐dose chemotherapy was administered in 4/21 (19%) patients with R0/R1 resection.

Among patients with inoperable disease, 4/11 (36%) received MC, 1/11 (9%) high‐dose chemotherapy, and none WAP‐RT.

### 3.1 Outcome

After a median (m‐) follow‐up of 37 (IQR 18–63) months, 32/38 (84%) patients had disease recurrence/progression, while 6/38 (16%) had not; 19/38 (50%) patients died of disease, 13/38 (34%) patients were alive with evidence of disease, and 6/38 (16%) alive with no evidence of disease.

**FIGURE 1:**
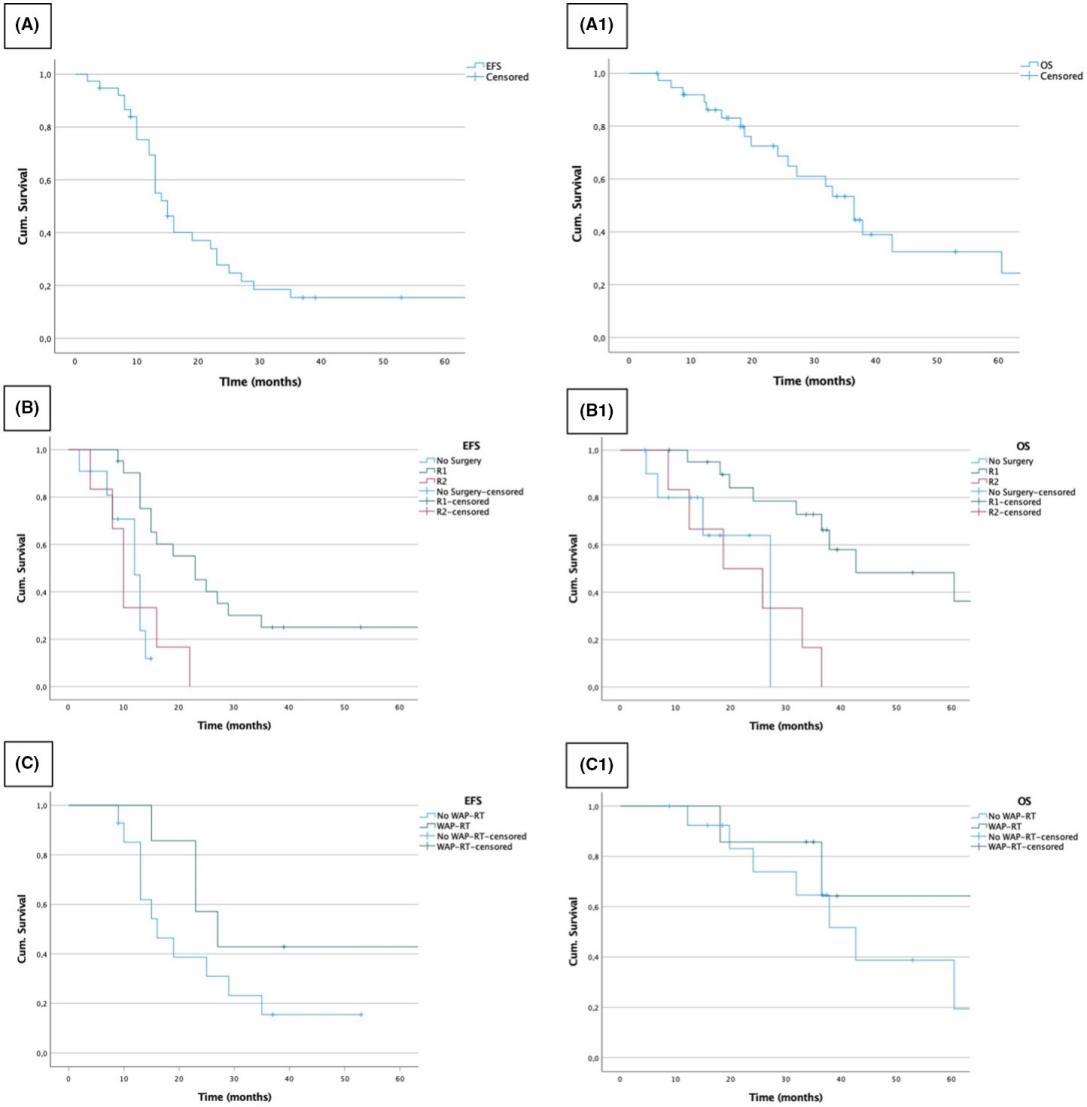
Survival curves. (A) Event‐free survival and (A1) overall survival of the whole population. (B) Event‐free survival and (B1) overall survival according to surgery. (C) Event‐free survival and (C1) overall survival according to whole abdominal‐pelvic radiation therapy (WAP‐RT) in patients who underwent R0/R1 resection.

Overall m‐EFS and m‐OS (95% CI) were 15 (13–17) and 37 (30–43) months, respectively (Figure 1‐A). All relapses/progressions occurred within 35 months from initial diagnosis. Figure S1‐A shows EFS and OS according to disease extent at diagnosis.

In 27 patients who underwent surgery, the m‐EFS and m‐OS (95% CI) were 19 (10–28) and 37 (30–43) months; 23 (14–32) and 43 (15–71) months after R0/R1 resection; and 10 (8–12) and 19 (3–35) months after R2 resection, respectively (Figure 1B). In 7/27 patients who received HIPEC, the m‐EFS and m‐OS (95% CI) were 19 (9–29) and 43 (33–52) months, respectively, while in 20/27 patients who did not have HIPEC, m‐EFS and m‐OS (95% CI) were 16 (8–24) and 37 (22–51) months, respectively (Figure S1B). In the subgroup of 7/21 R0/R1 patients who received WAP‐RT, m‐EFS and m‐OS (95% CI) were 27 (17–37) months and not reached, respectively, while in 14/21 R0/R1 patients who did not have WAP‐RT, m‐EFS and m‐OS (95% CI) were 16 (9–23) and 43 (29–56) months, respectively (Figure 1C). In the subgroup of 7/21 R0/R1 patients who received MC (5/7, treated also with WAP‐RT), m‐EFS and m‐OS (95% CI) were 25 (20–30) months and not reached, respectively, while in 14/21 R0/R1 patients who did not have MC, m‐EFS and m‐OS (95% CI) were 19 (10–28) and 43 (34–52) months, respectively (Figure S1C).

### 3.2 Matched‐pair analysis

**FIGURE 2:**
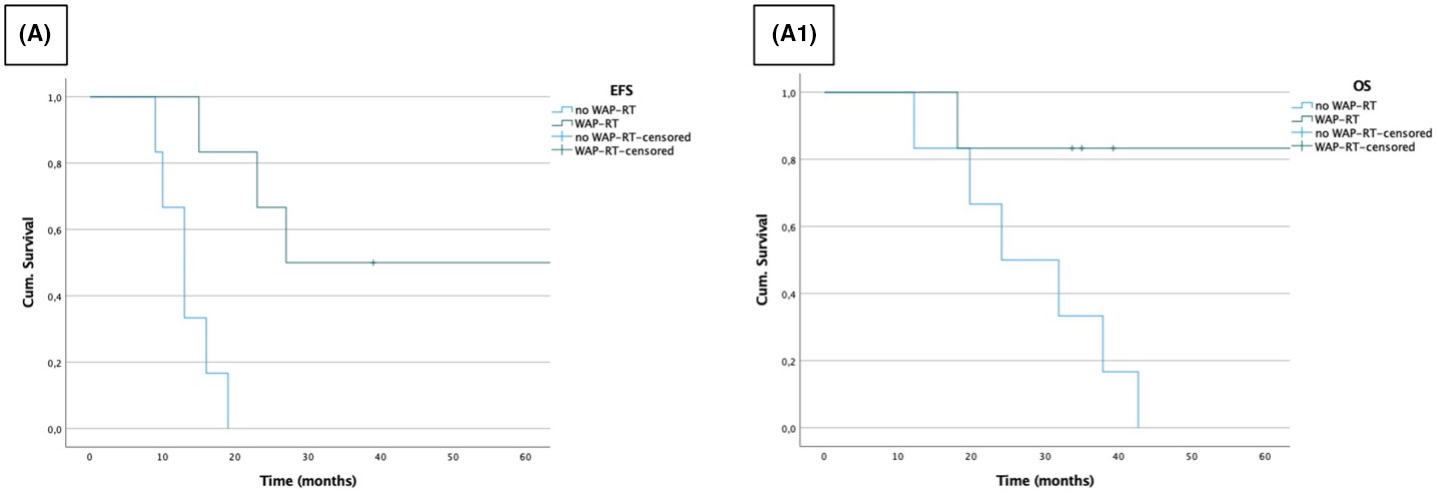
Survival curves of patients treated with whole abdominal‐pelvic radiation therapy (WAP‐RT). (A) Event‐free survival and (A1) overall survival in patients treated with and without WAP‐RT, matched for age, disease extent at diagnosis and R0/R1 resection.

We successfully paired eight of the nine patients who received WAP‐RT with a patient who did not present with the same disease extent at diagnosis and the same type of surgical resection. All the pairs but two had also comparable age at diagnosis. In this analysis the m‐EFS and m‐OS (95% CI) were 13 (8–18) and 32 (12–52) months in the no‐WAP‐RT group, respectively; 23 (12–34) months and not reached in the WAP‐RT group (Figure SS1‐D). Without considering the two couples not matched for age at diagnosis, leading to select the above mentioned 21 R0/R1 resected patients, m‐EFS and m‐OS (95% CI) in the no‐WAP‐RT group were 13 (range 9–19) and 24 (range 12–43) months, respectively. This exclusion did not affect the WAP‐RT group (estimates already mentioned above) (Figure 2).

The small number of patients coupled with variability of their characteristics and treatment combinations prevented additional matching for MC.

### 3.3 Long‐term survivors

LTS were all treated by multimodal therapy including R0/R1 resection surgery (5/21, [24%]). Median age at diagnosis was 32 (range 11–49) years. Disease extent at onset was intraperitoneal only in three patients (3/12 with peritoneal disease [25%]), peritoneal and subdiaphragmatic LN in two (2/10 [20%] with peritoneal and LN involvement), while none had liver or distant metastases. Best response to chemotherapy was PR in two patients, SD in one, not evaluable in two (adjuvant chemotherapy). Three patients received WAP‐RT (+ MC in 2/3), one high‐dose chemotherapy, none HIPEC. At the m‐follow‐up of 53 (IQR 39–64) months, no further event was observed. LTS were alive at 37, 39, 53, 64, and 209 months from primary diagnosis.

## 4 DISCUSSION

In this series of 38 patients treated over two decades at a single institution, five patients (15%) were LTS with an m‐follow‐up of 53 months. LTS were managed with a multimodal approach, including multiagent chemotherapy, which in a case included high‐dose chemotherapy, and macroscopic complete surgery. WAP‐RT was used in 3/5 LTS, followed by MC in 2/3, while none had HIPEC. LTS did not have metastases to the liver or above the diaphragm.

This is a small, retrospective, single‐institution study with all the limitations thereof. In particular, due to all the caveats of assessing radiologic and pathologic response retrospectively in soft tissue sarcoma, no correlation of response with the outcome could be assessed. In addition, the matched‐pair analysis could not expand the set of matching characteristics beyond a few that were judged to have a major prognostic impact. However, DSRCT is an ultra‐rare sarcoma in which prospective studies were never conducted and retrospective studies can generate hypothesis that can be then validated prospectively.^1^, ^2^

Our study confirms that DSRCT has a poor prognosis, with m‐OS of 37 months and <25% of patients alive at 5 years, as reported in previous series, in which m‐OS ranged from 17 to 60 months and 5‐year OS from 15% to 25%.^3^, ^8^, ^9^, ^10^, ^11^, ^16^, ^17^, ^20^, ^21^ However, LTS do exist, though their proportion is low, ranging from 3% to 33% (Table SS1). Comparison of results from available studies is challenged by their retrospective design, variation in length of follow‐up and, in most cases, lack of molecular confirmation of DSRCT diagnosis. The prognostic role of disease extent at diagnosis and complete surgical resection combined with multiagent chemotherapy is widely acknowledged.^3,6,11–15,21^ It is also well known that recurrence or progression is common in DSRCT even after complete cytoreductive surgery, mostly within the peritoneum, reflecting issues with achieving a complete removal of the microscopic residual tumor.^3^, ^10^, ^22^, ^23^ In essence, our study confirms these findings. In fact, our LTS had only localized disease and were all treated by multimodal therapy, including multiagent chemotherapy and complete surgical resection (R0/R1 resection). In our series, R0/R1 surgery correlated with a better OS and EFS compared to R2 or no surgery, adding to the notion that a macroscopic complete resection may be crucial. Despite that, 15/21 patients in the R0/R1 group relapsed. Of course, our data do not exclude that R2 resection can be considered in selected conditions with palliative intent.^24^, ^25^, ^26^ We also found that additional treatments (WAP‐RT and/or MC) after R0/R1 resection may improve survival, compared to R0/R1 surgery alone (data not shown). Given the retrospective nature of the study, we could not evaluate the impact of the prolonged multimodality approach received by the DSRCT patients treated within this study on their quality of life in the short and long term. However, this is certainly a sensitive that deserves to be investigated in future prospective studies.

Interestingly, in our study 3/5 LTS received WAP‐RT, followed by MC in 2/3. During the period covered by this retrospective study, the institutional policy differed between pediatric and adult patients. Due to limited data about the role of WAP‐RT and MC, in fact, these approaches in adult patients were usually not proposed. Therefore, our series cannot provide any result on the tolerability of WAP‐RT in patients above 18 years, that is indeed left to be assessed prospectively. The clinical relevance of postoperative WAP‐RT, which currently does not represent a standard treatment in DSRCT following a complete surgical resection, is described only in some series, with conflicting results (Table 2). Some patients had prolonged OS and progression‐free survival with WAP‐RT. Conversely, two studies^10^, ^23^ comparing outcome of patients treated with or without RT did not show a survival improvement and the authors suggested WAP‐RT only for selected patients within prospective clinical trials. Although small, our study would suggest a better prognosis when WAP‐RT, possibly combined with MC, is added to the treatment strategy following multiagent chemotherapy and complete surgical resection in patients presenting with peritoneal/LN disease. In fact, 3/7 patients who received multiagent chemotherapy plus R0/R1 surgical resection and adjuvant WAP‐RT (± MC) did not relapse, while 3/14 (one with a short follow‐up) did not recur among those treated with multiagent chemotherapy plus R0/R1 resection without adjuvant WAP‐RT or MC. In the subgroup of 7/21 R0/R1 patients who also received WAP‐RT (followed by MC in 5/7) m‐EFS was 27 months, with 3‐year EFS of 43%, while in 14/21 R0/R1 patients not treated with WAP‐RT, m‐EFS was 16 months, with 3‐year EFS of 16%. A matched‐pair analysis to compare patients treated and untreated with WAP‐RT that considered few characteristics (i.e., age, disease extent at diagnosis, and type of surgery) that may have a deeper impact on prognosis to make subgroups homogeneous, showed a potential benefit for WAP‐RT ± MC (m‐EFS 27 vs. 13 months in treated and untreated patients, respectively, with 3‐year EFS of 50% vs. 0%), suggesting that maximizing systemic and local treatments may pay off at least in selected patients with DSRCT. The potential activity of MC after R0/R1 resection has been described only in few case reports or small series.^3^, ^6^, ^27^ Scheer et al. showed that MC with metronomic chemotherapy consisting of cyclophosphamide/vinblastine after complete surgery correlated with prolonged time to relapse.^3^ Our study also suggests that MC could provide a survival benefit in R0/R1 patients. However, we could not discriminate between the prognostic effect of WAP‐RT and MC as a matched‐pair analysis was unfeasible due to number constraints. In terms of MC regimen, the combination of vinorelbine and low‐dose oral cyclophosphamide was the most used approach (8/12 [67%]) in our patients. In the lack of prospective data, this was essentially borrowed from the experience in another sarcoma type that affects children/young adults, that is, pediatric rhabdomyosarcoma.^28^ A prospective, larger study needs to be planned to shed light on this matter.

**TABLE 2:** List of papers reporting on WAP‐RT after surgery within multimodal treatment for DSRCT.

Authors, year	Number of patients treated with multimodal therapy (CT + CRS + WAP‐RT)	Molecular confirming test	Distant metastases at diagnosis	m‐FU (months)	Overall survival	Progression‐free survival
Goodman et al., 2002^33^	21/21 (100%)	NR	NR	28	m‐OS 32 months	m‐PFS 19 months
					3‐year OS 48%	3‐year PFS 19%
Lal et al., 2005^21^	29/66 (44%)	NR	27 (41%)	29	3‐year OS 55%	NR
Pinnix et al., 2012^34^	8/8 (100%)	NR	3 (38%)	31	NR	m‐PFS 9 months
Honoré et al., 2015^8^	4/38 (11%)	NR	18 (47%)	60	m‐OS 38 months	m‐PFS 16 months
Atallah et al., 2016^35^	27/103 (26%)	77%	5 (19%)	71	m‐OS 40 months	m‐PFS 23 months
					3‐year OS 63%	3‐year PFS 24%
Osborne et al., 2016^18^	32/32 (100%)	NR	15 (47%)	18	m‐OS 60 months	m‐PFS 12 months
					3‐year OS 64%	
Stiles et al., 2018^10^	21/102 (20%)	NR	6 (5%)	25	m‐OS 28 months	NR
					3‐year OS 29%	
Subbiah et al., 2018^23^	92/187 (49%)	NR	NR	NR	m‐OS 35 months	NR
					3‐year OS 48%	
Honoré et al., 2019^22^	26/100 (26%)	NR	25 (25%)	103	m‐OS 25 months	m‐PFS 11 months
					3‐year OS 35%	
Campos et al., 2020^36^	4/19 (21%)	21%	11 (58%)	95	m‐OS 27 months	m‐PFS 9 months
					3y‐OS 38%	
Liu et al., 2021^30^	6/6 (100%)	100%	5 (83%)	47	2‐year OS 75%	2‐year PFS 75%

Abbreviations: CT, chemotherapy; CRS, cytoreductive surgery; FU, follow‐up; m‐, median; NR, not reported; OS, overall survival; PFS, progression‐free survival; WAP‐RT, whole abdomino‐pelvic radiation therapy.

Consistent with most available literature,^22^, ^23^, ^29^ HIPEC did not result in any survival benefit in our cohort. Conversely, in the study by Hayes‐Jordan,^13^ R0/R1 surgery and HIPEC significantly improved m‐OS. However, the outcome differences may well be attributed to case selection and completeness of surgical resection. Finally, in a recent phase II trial^17^ of 14 DSRCT treated with chemotherapy, R0/R1 surgery plus HIPEC, the combination of aggressive local therapies appeared to be beneficial in local control, but WAP‐RT was incorporated as well.

The most effective chemotherapeutic regimen to consider in primary DSRCT is left to be defined. The most commonly used combinations contain anthracyclines, ifosfamide/cyclophosphamide, cisplatin, and etoposide^3^, ^11^ Patients in our study received regimens based on anthracyclines and ifosfamide. Irinotecan was instead given only in two cases (already reported by Ferrari et al.^6^) in combination with ifosfamide, vincristine and actinomycin‐D (IrIVA), with short‐interval regimens. These patients relapsed at 21 and 27 months from diagnosis. Also Liu et al.^30^ reported that 3/6 patients treated with irinotecan‐containing regimens, R1 surgery, and WAP‐RT were alive and disease‐free at 21, 47, and 60 months (m‐follow‐up 47 months). Concurrently, we assessed the activity of irinotecan in combination with trabectedin in a new patient‐derived xenograft (PDX) model of DSRCT observing a complete response to this regimen. Finally, we saw irinotecan plus trabectedin stabilizing the disease in two patients with advanced DSRCT who failed two previous treatment lines.^31^ Although very preliminary, these results suggest incorporating irinotecan in the first‐line regimen for DSRCT. Indeed, a novel dose‐density combination strategy using irinotecan has been recently described in high‐risk sarcomas such as DSRCT.^30^, ^32^ Of note, vinorelbine also showed major antitumor effect in the DSRCT PDX model (submitted data).

In conclusion, with all the aforementioned limitations, our findings show that some patients with DSRCT become LTS, essentially if having a disease limited to the abdominal‐pelvic region (peritoneum/LN). Complete surgery seems to play a crucial role while the addition of WAP‐RT +/− MC to R0/R1 resection deserves further investigation. Incorporating irinotecan in the primary disease treatment also deserves further evaluation.

The retrospective nature of this analysis and the small number of patients do not allow to generate strong recommendations, but rather suggest hypotheses in order to guide new further studies. Indeed, prospective studies in homogeneous patient populations on integrated approaches maximizing local control are needed in DSRCT patients with abdominal‐pelvic extent. Besides, new systemic agents are urgently needed to improve the prognosis of this ultra‐rare aggressive disease.

## AUTHOR CONTRIBUTIONS

**Claudia Giani:** Data curation (equal); project administration (equal); writing – original draft (equal); writing – review and editing (equal). **Stefano Radaelli:** Data curation (equal); writing – original draft (equal); writing – review and editing (equal). **Rosalba Miceli:** Formal analysis (equal); methodology (equal); writing – original draft (equal); writing – review and editing (equal). **Lorenza Gandola:** Supervision (equal); writing – original draft (equal); writing – review and editing (equal). **Claudia Sangalli:** Supervision (equal); writing – original draft (equal); writing – review and editing (equal). **Anna Maria Frezza:** Supervision (equal); writing – original draft (equal); writing – review and editing (equal). **Salvatore Provenzano:** Data curation (supporting); writing – review and editing (equal). **Sandro Pasquali:** Supervision (equal); writing – original draft (equal); writing – review and editing (equal). **Rossella Bertulli:** Data curation (supporting); writing – review and editing (equal). **Marco Fiore:** Data curation (supporting); writing – review and editing (equal). **Dario Callegaro:** Data curation (supporting); writing – review and editing (equal). **Michela Casanova:** Data curation (equal); writing – original draft (equal); writing – review and editing (equal). **Stefano Chiaravalli:** Data curation (supporting); writing – review and editing (equal). **Paola Collini:** Data curation (equal); writing – original draft (equal); writing – review and editing (equal). **gianpaolo dagrada:** Data curation (equal); writing – original draft (equal); writing – review and editing (equal). **Carlo Morosi:** Data curation (equal); writing – review and editing (equal). **Nadia Zaffaroni:** Data curation (equal); supervision (equal); writing – original draft (equal); writing – review and editing (equal). **Paolo G. Casali:** Conceptualization (equal); methodology (equal); supervision (equal); writing – review and editing (equal). **Andrea Ferrari:** Data curation (equal); writing – original draft (equal); writing – review and editing (equal). **Alessandro Gronchi:** Conceptualization (equal); data curation (equal); methodology (equal); supervision (equal); writing – original draft (equal); writing – review and editing (equal). **Silvia Stacchiotti:** Conceptualization (equal); data curation (equal); methodology (equal); supervision (equal); writing – original draft (equal); writing – review and editing (equal).

## FUNDING INFORMATION

This research did not receive any specific grant from funding agencies in the public, commercial, or not‐for‐profit sectors.

## CONFLICT OF INTEREST STATEMENT

None of the authors has any interest to report directly related to this manuscript.

*Outside the scope of this manuscript*:

Frezza reports Institutional research funds from: Advenchen Laboratories, Amgen Dompé, AROG Pharmaceuticals, Bayer, Blueprint Medicines, Daiichi Sankyo, Deciphera, Eisai, Eli Lilly, Epizyme Inc, GlaxoSmithKline, Karyopharm Pharmaceuticals, Novartis, Pfizer, PharmaMar, SpringWorks.Provenzano reports Institutional research funds from: Advenchen Laboratories, Amgen Dompé, AROG Pharmaceuticals, Bayer, Blueprint Medicines, Daiichi Sankyo, Deciphera, Eisai, Eli Lilly, Epizyme Inc, GlaxoSmithKline, Karyopharm Pharmaceuticals, Novartis, Pfizer, PharmaMar, SpringWorks.

Bertulli reports Institutional research funds from: Advenchen Laboratories, Amgen Dompé, AROG Pharmaceuticals, Bayer, Blueprint Medicines, Daiichi Sankyo, Deciphera, Eisai, Eli Lilly, Epizyme Inc, GlaxoSmithKline, Karyopharm Pharmaceuticals, Novartis, Pfizer, PharmaMar, SpringWorks.Casali reports Institutional research funds from: Advenchen Laboratories, Amgen Dompé, AROG Pharmaceuticals, Bayer, Blueprint Medicines, Daiichi Sankyo, Deciphera, Eisai, Eli Lilly, Epizyme Inc, GlaxoSmithKline, Karyopharm Pharmaceuticals, Novartis, Pfizer, PharmaMar, SpringWorks. Honoraria for speaker, consultancy or advisory role from: Bayer, Deciphera, Eisai, Eli Lilly, Pfizer.

Stacchiotti reports Institutional research funds from: Advenchen Laboratories, Amgen Dompé, AROG Pharmaceuticals, Bayer, Blueprint Medicines, Daiichi Sankyo, Deciphera, Eisai, Eli Lilly, Epizyme Inc, GlaxoSmithKline, Karyopharm Pharmaceuticals, Novartis, Pfizer, PharmaMar, SpringWorks. Honoraria, consultancy or advisory role from: Bayer, Bavarian Nordic, Deciphera, Eli Lilly, Epizyme Inc., Daiichi Sankyo Pharma, GlaxoSmithKline, Maxivax, PharmaMar. Travel, accommodations, expenses from: PharmaMar.

The authors report no other conflicts of interest.

## Supporting information


**Table S1** List of papers available since 1996 reporting DSRCT event‐free long‐term survivors and their characteristics.
**Table S2** Maintenance chemotherapy.
**Table S3** Patients treated with whole abdomino‐pelvic radiation therapy.
**Table S4** Chemotherapy regimens received in first‐line and best response.
**Figure S1** (A) Event‐free survival and (A1) overall survival according to disease extent at diagnosis. (B) Event‐free survival and (B1) overall survival according to HIPEC in patients treated with surgery. (C) Event‐free survival and (C1) overall survival according to maintenance CT in R0/R1 patients. (D) Event‐free survival and (D1) overall survival in patients treated with and without WAP‐RT, matched for disease extent at diagnosis and type of surgery (R0/R1 or R2 resection). CT, chemotherapy; EFS, event‐free survival; HIPEC, hyperthermic intraperitoneal chemotherapy; OS, overall survival; WAP‐RT, whole abdominopelvic radiation therapy.
