# Supplementary material for: Long‐term survivors with desmoplastic small round cell tumor (DSRCT): Results from a retrospective single‐institution case series analysis
Source: Cancer Med. 2023 Mar 23;12(9):10694–703. doi: 10.1002/cam4.5829 (PMC10225189; doi:10.1002/cam4.5829)
Supplement: Supplementary file 1 — Table S1 List of papers available since 1996 reporting DSRCT event‐free long‐term survivors and their characteristics. Table S2 Maintenance chemotherapy. Table S3 Patients treated with whole abdomino‐pelvic radiation therapy. Table S4 Chemotherapy regimens received in first‐line and best response. Figure S1 (A) Event‐free survival and (A1) overall survival according to disease extent at diagnosis. (B) Event‐free survival and (B1) overall survival according to HIPEC in patients treated with surgery. (C) Event‐free survival and (C1) overall survival according to maintenance CT in R0/R1 patients. (D) Event‐free survival and (D1) overall survival in patients treated with and without WAP‐RT, matched for disease extent at diagnosis and type of surgery (R0/R1 or R2 resection). CT, chemotherapy; EFS, event‐free survival; HIPEC, hyperthermic intraperitoneal chemotherapy; OS, overall survival; WAP‐RT, whole abdominopelvic radiation therapy. [file CAM4-12-10694-s001.docx]

**Supplementary Table 1** List of papers available since 1996 reporting DSRCT event-free long-survivors and their characteristics.

| **Author, year** | **# of patients** | **Event-free long-survivors* (%)** | **# of metastatic cases at diagnosis** | **CT + Surgery + WAP-RT** | **Outcome** | **Months from diagnosis in patients alive and disease-free** |
| --- | --- | --- | --- | --- | --- | --- |
| Kushner et al, 1996^1^ | 12 | 1 | No | No  (Local RT) | NR | 39 |
| Kretschmar et at, 1996^2^ | 101 | 2 | NR | 2/2 | m-OS 17 mos | 40, 48 |
| Kurre et al, 2000^3^ | 3 | 2 | 1/2 | No  (Local RT) | NA | 42, 66 |
| Hassan, 2005^4^ | 12 | 1 | No | No  (Local RT) | m-OS 34 mos | 36 |
| Lal et al, 2005^5^ | 66 | 4 | NR | 3/4 (75%) | 3y-OS 44% | 68, 94, 121, 134  (m-FU 29 mos) |
| Saab et al, 2007^6^ | 11 | 2 | 1/2 | No  (Local RT) | m-OS 21 mos | 96, 120 |
| Bisogno et al, 2010^7^ | 14 | 1/14 | No | No  (No RT) | m-OS 24 mos  3y-OS 39% | 50  (m-FU 27 mos) |
| Zhang et al, 2014^8^ | 11 | 2 | No | No  (No RT) | m-OS 24 mos | 36, 42 |
| Honoré et al, 2015^9^ | 38 | 1 | NR | 1/1 | m-OS 38 mos | 37  (m-FU 60 mos) |
| Zhang et al, 2015^10^ | 11 | 2 | 2/2 | No  (Local RT) | m-OS 29 mos | 40, 72 |
| Bailey et al, 2018^11^ | 4 | 2 | 1/2 | 1/2 | NA | 60, 91 |
| Honoré et al, 2019^12^ | 100 | 5 | No | 4/5 | m-OS 25 mos | >60  (m-FU 103 mos) |
| Scheer et al, 2019^13^ | 60 | 2 | NR | No  (No RT) | m-OS 24 mos | 66, 73  (m-FU 38 mos) |
| Xiao et al, 2021^14^ | 1 | 1 | Yes | Yes | NA | 72 |
| Liu et al, 2021^15^ | 6 | 2 | 2/2 | 2/2 | NR | 47, 60  (m-FU 47 mos) |

Legend: NA = not applicable; NR = not reported; CT = chemotherapy; RT = radiation therapy; WAP-RT = whole abdominopelvic radiation therapy; m- = median; OS = overall survival; mos = months; y = year, FU = follow-up. * = defined as patients with no evidence of disease > 36 months.

**Supplementary Table 2** Maintenance chemotherapy

| **Maintenance chemotherapy** | N (%) |
| --- | --- |
| Etoposide | 2/12 (17%) |
| Cyclophosphamide | 1/12 (8%) |
| Vinorelbine + cyclophosphamide | 8/12 (67%) |
| Irinotecan/Temozolomide | 1/12 (8%) |

**Supplementary Table 3** Patients treated with whole abdomino-pelvic radiation therapy

| **Patient, sex** | **Age at diagnosis (years)** | **Stage at diagnosis** | **WAP-RT total dose (Gy) / fraction dose (Gy)** | **Technique** | **EFS (months)** |
| --- | --- | --- | --- | --- | --- |
| 1, M | 23 | Peritoneal disease + sub-diaphragmatic LN | 25,5 / 1.5 | VMAT | 27 |
| 2, M | 18 | Supra-diaphragmatic disease | 25,5 / 1.5 | VMAT | 23 |
| 3, M | 11 | Multiple peritoneal nodules | 30 / 1.5 | VMAT | 39 |
| 4, M | 17 | Peritoneal disease + sub-diaphragmatic LN | 30 / 1.5 | 3DCRT | 64 |
| 5, M | 32 | Multiple peritoneal nodules | 32 | NA | 209 |
| 6, M | 7 | Multiple peritoneal nodules | 15 / 1.5 | 3DCRT | 23 |
| 7, M | 13 | Peritoneal disease + sub-diaphragmatic LN | 30 / 1.5 | 3DCRT | 16 |
| 8, M | 18 | Liver metastases | 15 / 1.5 | 3DCRT | 15 |
| 9, F | 10 | Peritoneal disease + sub-diaphragmatic LN | 25 / 1.5 | IMRT (7 beams) | 4 |

Legend: M = male; F = female; 3DCRT = 3dimensional conformal radiation therapy; LN = lymph nodes; IMRT = intensity-modulated radiation therapy; VMAT = volumetric modulated arc therapy; EFS = event-free survival; WAP-RT = whole abdomino-pelvic radiation therapy; NA = not aveilable.

**Supplementary Table 4** Chemotherapy regimens received in first-line and best response.

| **First-line chemotherapy** | **Treated patient # (%)** | **Response by RECIST 1.1** |
| --- | --- | --- |
| VAI - IE | 12/38 (32%) | PR 5/12 (42%)  SD 3/12 (25%)  NE 4/12 (33%) |
| IVADo regimens  IVADo  IVADo/IVA  IVADo/IVE  IVADo/ICE | 5/38 (13%)  2/38 (5%)  1/38 (3%)  1/38 (3%)  1/38 (3%) | PR 1/2 (50%), NE 1/2 (50%)  PR 1/1 (100%)  PR 1/1 (100%)  SD 1/1 (100%) |
| EI-PE | 4/38 (11%) | PR 1/4 (25%)  SD 2/4 (50%)  NE 1/4 (25%) |
| Adriamycin/ifosfamide - PE | 3/38 (7%) | SD 3/3 (100%) |
| VAI - PE | 3/38 (7%) | SD 2/3 (67%)  NE 1/3 (33%)* |
| VAC - IE | 3/38 (7%) | PR 2/3 (67%)  SD 1/3 (33%) |
| IrIVA^6^ | 2/38 (5%) | PR 2/2 (100%) |
| VAIA | 1/38 (3%) | PD 1/1 (100%) |
| Anthracycline/ vincristine/ ifosfamide – vincristine/ cyclophosphamide/ actinomycin-D | 1/38 (3%) | PR 1/1 (100%) |
| EI | 1/38 (3%) | PR 1/1 (100%) |
| EI - IE | 1/38 (3%) | PR 1/1 (100%) |
| CEVAIE^16^ | 1/38 (3%) | NE 1/1 (100%) |
| IVE | 1/38 (3%) | SD 1/1 (100%) |

Legend: VAI = vincristine, adriamycin, ifosfamide; EI = epirubicin, ifosfamide; IE = ifosfamide, etoposide; PE = cisplatin, etoposide; VAI-IE = vincristine, adriamycin, ifosfamide, etoposide; VAI-PE = vincristine, adriamycin, ifosfamide, cisplatin, etoposide (* = epirubicin instead of adriamycin); VAC-IE = vincristine, adriamycin, cyclophosphamide, ifosfamide, etoposide; IVA = ifosfamide, vincristine, actinomycin-D; IVE = ifosfamide, vincristine, etoposide; ICE = ifosfamide, carboplatin, etoposide; IrIVA^6^ = irinotecan, ifosfamide, vincristine, actinomycin-D; IVADo= ifosfamide, vincristine, actinomycin-D, doxorubicin; VAIA = vincristine, adriamycin, ifosfamide, actinomycin-D; CEVAIE = ifosfamide, vincristine, actinomycin-D, carboplatin, epirubicin, etoposide; PR = partial response; SD = stable disease; PR progressive disease; NE= not evaluable.

**Supplementary Figure 1**

**
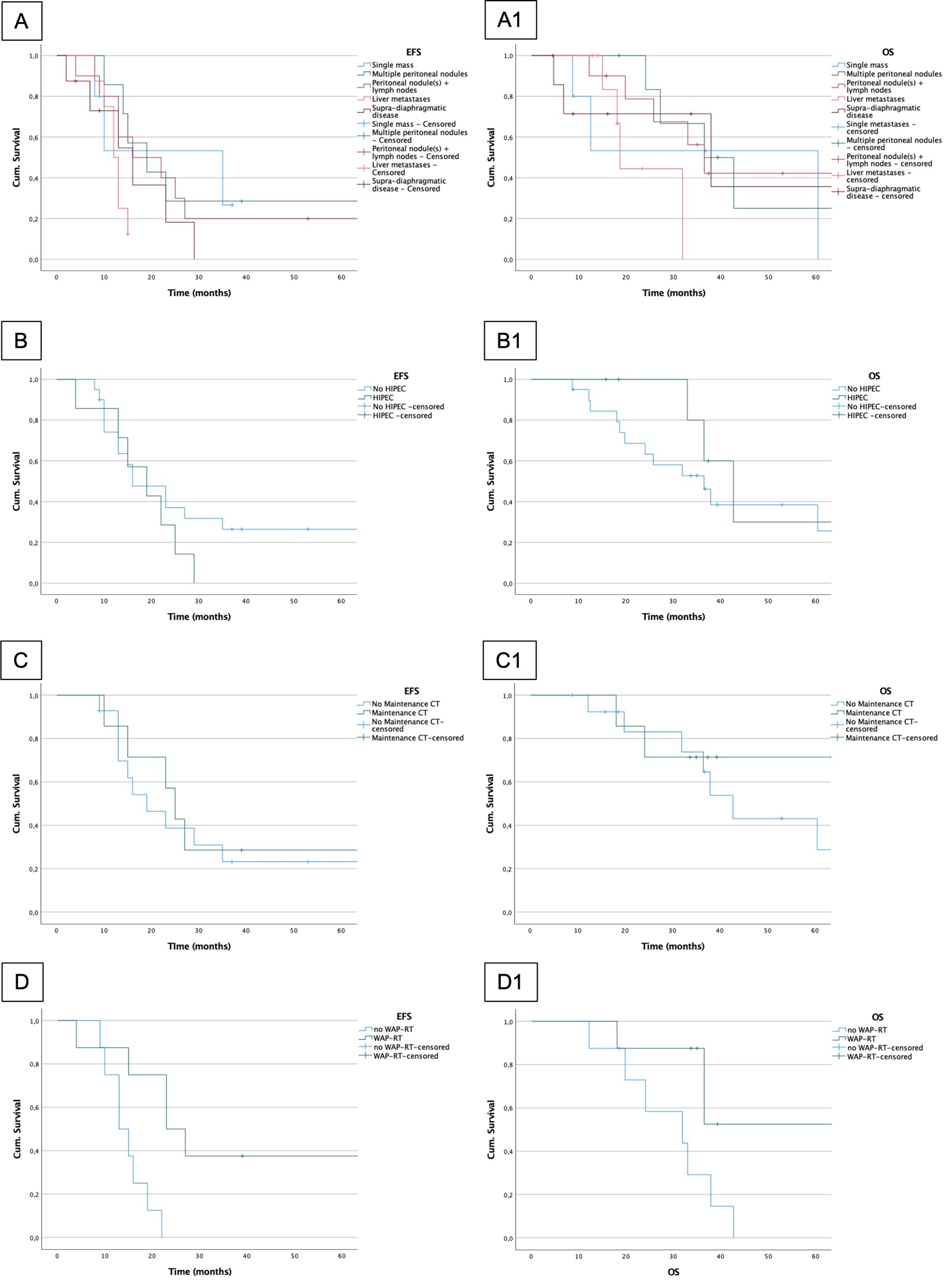
**

**Supplementary Figure 1 A** Event free survival and (A1) overall survival according to disease extent at diagnosis. **B** Event free survival and (B1) overall survival according to HIPEC in patients treated with surgery. **C** Event free survival and (C1) overall survival according to maintenance CT in R0/R1 patients. **D** Event free survival and (D1) overall survival in patients treated with and without WAP-RT, matched for disease extent at diagnosis and type of surgery (R0/R1 or R2 resection).

Legend: EFS = event-free survival; OS = overall survival; CT = chemotherapy; HIPEC = hyperthermic intraperitoneal chemotherapy; WAP-RT = whole abdominopelvic radiation therapy.

References

1. Kushner BH, LaQuaglia MP, Wollner N, et al. Desmoplastic small round-cell tumor: prolonged progression-free survival with aggressive multimodality therapy. *J Clin Oncol*. 1996;14(5):1526-1531.

2. Kretschmar CS, Colbach C, Bhan I, Crombleholme TM. Desmoplastic small cell tumor: a report of three cases and a review of the literature. *J Pediatr Hematol Oncol*. 1996;18(3):293-298.

3. Kurre P, Felgenhauer JL, Miser JS, Patterson K, Hawkins DS. Successful dose-intensive treatment of desmoplastic small round cell tumor in three children. *J Pediatr Hematol Oncol*. 2000;22(5):446-450.

4. Hassan I, Shyyan R, Donohue JH, et al. Intraabdominal desmoplastic small round cell tumors: a diagnostic and therapeutic challenge. *Cancer*. 2005;104(6):1264-1270.

5. Lal DR, Su WT, Wolden SL, Loh KC, Modak S, La Quaglia MP. Results of multimodal treatment for desmoplastic small round cell tumors. *J Pediatr Surg*. 2005;40(1):251-255.

6. Saab R, Khoury JD, Krasin M, Davidoff AM, Navid F. Desmoplastic small round cell tumor in childhood: the St. Jude Children’s Research Hospital experience. *Pediatr Blood Cancer*. 2007;49(3):274-279.

7. Bisogno G, Ferrari A, Rosolen A, et al. Sequential intensified chemotherapy with stem cell rescue for children and adolescents with desmoplastic small round-cell tumor. *Bone Marrow Transplant*. 2010;45(5):907-911.

8. Zhang G, Liu G, Zhao D, Cui X, Li G. Desmoplastic small round cell tumor of the abdomen and pelvis: clinicopathological characters of 12 cases. *ScientificWorldJournal*. 2014;2014:549612.

9. Honoré C, Amroun K, Vilcot L, et al. Abdominal desmoplastic small round cell tumor: multimodal treatment combining chemotherapy, surgery, and radiotherapy is the best option. *Ann Surg Oncol*. 2015;22(4):1073-1079.

10. Zhang S, Zhang Y, Yu YH, Li J. Results of multimodal treatment for desmoplastic small round cell tumor of the abdomen and pelvis. *Int J Clin Exp Med*. 2015;8(6):9658-9666.

11. Bailey K, Roth M, Weiser D, Gill J. High-Dose Chemotherapy with Stem Cell Rescue in Desmoplastic Small Round Cell Tumor: A Single-Institution Experience and Review of the Literature. *Sarcoma*. 2018;2018:1948093.

12. Honoré C, Delhorme JB, Nassif E, et al. Can we cure patients with abdominal Desmoplastic Small Round Cell Tumor? Results of a retrospective multicentric study on 100 patients. *Surg Oncol*. 2019;29:107-112.

13. Scheer M, Vokuhl C, Blank B, et al. Desmoplastic small round cell tumors: Multimodality treatment and new risk factors. *Cancer Med*. 2019;8(2):527-542.

14. Xiao J, Browning MB, Boyd KP, et al. Multimodal Therapy Including Cytoreductive Surgery and Hyperthermic Intraperitoneal Chemotherapy Can Result in Long-term Disease-free Survival in Pediatric Desmoplastic Small Round Cell Tumor With Extraperitoneal Disease. *J Pediatr Hematol Oncol*. 2021;43(6):228-231.

15. Liu KX, Collins NB, Greenzang KA, et al. The use of interval-compressed chemotherapy with the addition of vincristine, irinotecan, and temozolomide for pediatric patients with newly diagnosed desmoplastic small round cell tumor. *Pediatr Blood Cancer*. 2020;67(10):e28559.

16. Klingebiel T, Boos J, Beske F, et al. Treatment of children with metastatic soft tissue sarcoma with oral maintenance compared to high dose chemotherapy: report of the HD CWS-96 trial. *Pediatr Blood Cancer*. 2008;50(4):739-745.
